# Involvement of stem cell factor and c-kit in corneal wound healing in mice

**Published:** 2012-06-07

**Authors:** Kazuhisa Miyamoto, Takeshi Kobayashi, Yasuhito Hayashi, Yuan Zhang, Yuko Hara, Masakatsu Higashine, Atsushi Shiraishi, Yuichi Ohashi

**Affiliations:** 1 Department of Ophthalmology, Ehime University Graduate School of Medicine, Shitsukawa, Toon, Ehime, Japan; 2 Department of Ophthalmology and Regenerative Medicine, Ehime University Graduate School of Medicine, Shitsukawa, Toon, Ehime, Japan; 3 Department of Stem Cell Biology, Ehime University Graduate School of Medicine, Shitsukawa, Toon, Ehime, Japan; 4 Department of Infectious Diseases, Ehime University Graduate School of Medicine, Shitsukawa, Toon, Ehime, Japan

## Abstract

**Purpose:**

To study the roles played by stem cell factor (SCF) and SCF receptor c-kit in wound healing of corneal epithelial cells.

**Methods:**

A 2 mm corneal epithelial wound was made in control (WBB6F1^+/+^), SCF (Sl/Sl^d^)-, and c-kit (W/W^v^) mutant mice, and the speed of wound healing, 5-bromo-2’-deoxyuridine (BrdU) incorporation, and scanning electron microscopic (SEM) morphology of the corneas were examined. The incorporation of BrdU and the degree of cell attachment in cultured mouse corneal epithelial cells (MCECs) isolated from WBB6F1^+/+^, Sl/Sl^d^, and W/W^v^ mice were examined. Cultured immortalized human corneal epithelial cells (HCECs) were examined by a cell attachment assay after their exposure to anti-SCF antibodies, tyrosine kinase inhibitor (genistein), and competitive Arg-Gly-Asp (RGD) peptide, as well as on cultures treated with extracellular matrix.

**Results:**

The speed of corneal wound healing was slower in Sl/Sl^d^ and W/W^v^ mice than in controls (p<0.01) and the speed of healing in Sl/Sl^d^ mice recovered after topical application of SCF (8 ng/ml). No significant difference was found in the BrdU incorporation assay either in vivo or in vitro. Loosened epithelial cells were detected at wound margins in W/W^v^ mice by SEM. The cell attachment rate was increased by 157% in cells from WBB6F1^+/+^ and 252% in Sl/Sl^d^ MCECs by recombinant mouse SCF; however, no significant difference was found in W/W^v^ MCECs. Anti-SCF antibodies (Ab), genistein, and RGD peptide reduced the percentage of attached HCECs. Anti-SCF Ab inhibited the attachment of HCECs on fibronectin, laminin, or type IV collagen coated dishes.

**Conclusions:**

These findings indicate that the SCF/c-kit system may play a role in corneal wound healing through epithelial cell attachment.

## Introduction

Stem cell factor (SCF), also called c-kit ligand, steel factor, and mast cell growth factor, is composed of 164 amino acids and has a molecular weight of 30 kDa. It exists in soluble and membrane-bound forms [1-4]. SCF signals are transmitted by the c-kit receptor, which belongs to the same subfamily of tyrosine kinases receptors as platelet-derived growth factor (PDGF) and granulocyte macrophage colony-stimulating factor (GM-CSF) [2-5]. c-kit has an immunoglobulin-like structure in the extracellular domain and a tyrosine kinase-like structure in the cytoplasmic domain. The tyrosine kinase activity of this receptor is tightly regulated by SCF and is known to play a crucial role in signal transduction pathways involved in the growth and differentiation of various cells [6-10]. c-kit is distributed in such tissues as bone marrow, spleen, thymus, skin, and testis, while SCF is expressed in placental tissue, bone marrow stromal cells, venous endothelial cells, fibroblasts, and Sertoli cells [11-13]. The SCF/c-kit system functions mainly in the stimulation and maturation of myeloid, erythroid, and lymphoid progenitors, and in the differentiation and growth of melanocytes, germ cells, and mast cells [6,9,10,14-16].

Recent studies have demonstrated that epithelial cells express SCF and/or c-kit and the SCF/c-kit system has important functional roles in epithelial cells. Thus, ovarian surface epithelial cells express SCF and c-kit, suggesting that they are involved in normal ovarian surface epithelial biology as well as ovarian cancer [17]. In the skin, SCF and c-kit are expressed in mast cells, melanocytes, and epithelial cells, and they are involved in epithelial wound healing, melanocyte proliferation and migration, and hair cycling [18-20]. The SCF/c-kit system is also involved in the regenerative processes in the liver [21].

However, there have been only three studies that have examined the SCF in ocular tissues: infiltrating fibroblasts in pterygia, choroidal melanocytes, and iris pigment epithelial cells [22-24]. However, the localization and function of the SCF/c-kit system in ocular surface tissues are still undetermined.

The SCF is located at the steel (*Sl*) locus on chromosome 12 in humans and on chromosome 10 in mice, and c-kit at the white spotting (*W*) locus of chromosome 4 in humans and on mouse chromosome 5 [25-29]. A complete loss of SCF or c-kit function is lethal, but mice with mutations at either of these alleles (Sl/Sl^d^ and W/W^v^) show similar defects in hematopoiesis, gametogenesis, and melanogenesis [30-32]. Mice with mutations at either locus typically have unpigmented coats, are sterile, and are deficient in erythrocytes and mast cells, as well as the precursors for multiple hematopoietic lineages. Thus, these mice have been used to investigate the SCF/c-kit system [12,30,31,33,34].

The purpose of this study was to determine the localization of SCF and c-kit in the mouse cornea, and to examine what role they play in corneal epithelial wound healing. To accomplish this, we first investigated corneal wound healing in both Sl/Sl^d^- and W/W^v^–mutant mice in vivo. We also examined cultured mouse corneal epithelial cells isolated from Sl/Sl^d^ and W/W^v^ mice.

## Methods

### Animals

We used WBB6F1-Sl/Sl^d^ and [29] WBB6F1-W/W^v^ [28] mice and WBB6F1^+/+^ mice for control. Genotypes of mutant mice were determined by SLC, Inc. (Hamamatsu, Japan). All of the mice were purchased from SLC, Inc. and housed in conformity with the Statement on the Use of Animals at Ehime University School of Medicine. Mice were gender-matched for the experiments and used at 6 to 10weeks of age. All mice were handled in accordance with the guidelines in the ARVO Statement for the Use of Animals in Ophthalmic and Vision Research.

### Measurement of corneal wound healing in SCF and C-kit mutant mice

Mice were anesthetized by an intraperitoneal injection of phenobarbital (50 mg/kg). There were 5 animals in each group, viz., the SCF mutant (Sl/Sl^d^), c-kit mutant (W/W^v^), and control WBB6F1-^+/+^ mice. A 2 mm central corneal epithelial defect was made on one eye with an excimer laser (EC-5000; NIDEK, Shizuoka, Japan), and the depth of wound was standardized at 30 µm. Corneas were stained with 0.4% methylene blue in phosphate-buffered saline (PBS), and the injured area was photographed at 6, 12, 18, 24, 30, 36, 42, and 48 h after creating the epithelial injury. The size of the injured area was measured on the images by Canvas™ ver 6.0 (Deneba software, Miami, FL).

### In vivo 5-bromo-2’-deoxyuridine (BrdU) incorporation

In addition to assessing the degree of wound healing, animals (n=6 for each genotype and for each time point) were injected intraperitoneally with 8 mg/µl (200 µg/g bodyweight) of 5-bromo-2’-deoxyuridine (Sigma-Aldrich, St. Louis, MO) in PBS 2 h before euthanasia.

### RNA extraction and reverse transcription-polymerase chain reaction (RT–PCR)

Total RNA was extracted from the corneas excised from WBB6F1^+/+^ mice using the RNeasy kit (Qiagen, Valencia, CA) and then reverse-transcribed using Omniscript Reverse Transcriptase (Qiagen) according to the manufacturer’s protocol. The extracts were divided into tubes containing PCR reaction mixture (AmpliTaq Gold; Applied Biosystems, Inc., Tokyo, Japan) with specific primers (Table 1).

**Table 1:** Sequences of primers used in RT–PCR.

**Primer**	**Sequence (5′-3′)**	**Product size (bp)**	**GenBank number**
SCF	F-TCATGGTGCACCGTATCCTA	170 bp	NM_013598
	R-CCTTGGCATGTTCTTCCACT		
c-kit	F-GGGCTAGCCAGAGACATCAG	160 bp	NM_001122733
	R-AGGAGAAGAGCTCCCAGAGG		NM_021099
β-actin	F-CCTGTATGCCTCTGGTCGTA	260 bp	NM_007393
	R-CCATCTCCTGCTCGAAGTCT		

The mixture was amplified with the TaKaRa PCR Thermal Cycler (TAKARA BIO Inc., Otsu, Japan): 40 cycles of 30 s at 94 °C, 30 s at 58 °C, and 1 min at 72 °C, followed by extension at 72 °C for 7 min. Then 5 µl of this solution was electrophoresced on 1.5% agarose gel and stained with 1 µg/ml of ethidium bromide and viewed with a transilluminator.

### Immunohistochemistry

For immunostaining of SCF and c-kit, corneas were fixed in periodate-lysine-paraformaldehyde containing 4% sodium metaperiodate for 30 min and placed in 10% sucrose overnight. Samples were frozen and cut into 7 µm sections. For immunostaining for BrdU, eyes were fixed in 4% paraformaldehyde in PBS overnight and embedded in paraffin. Then 5 µm sections were cut, deparaffinized, and rehydrated. After the endogenous peroxidase was blocked with 0.3% hydrogen peroxidase for 20 min, sections were incubated with anti-mouse c-kit rabbit IgG (0.5 µg/ml; Santa Cruz, Santa Cruz, CA), anti-mouse SCF rabbit IgG (1.3 µg/ml, Genzyme, Cambridge, MA), or anti-BrdU monoclonal antibody (0.5 µg/ml, Lab Vision, Fremont, CA) for 24 h at 4 °C. The sections were developed with the peroxidase-DAB method using PAPKIT™ (ZYMED, San Francisco, CA) or M.O.M.™ immunodetection kits (Vector Laboratories, Burlingame, CA) according to the manufacturer’s instructions. Isotype-matched, irrelevant antibodies were used to rule out false positives for each immunostained group.

### Topical instillation of recombinant SCF on injured corneas

A 2 mm corneal epithelial defect was created on one eye of each mouse of the three groups as described above, and 8 ng/ml of recombinant mouse SCF (rmSCF, Genzyme) in 5 µl of PBS was applied every 6 h for up to 48 h. As control, an identical number of mice were given 5 µl of PBS-BSA (BSA) of the same protein concentration as the rmSCF. The injured corneas were photographed every 6 h and size of the injured area was measured as described above.

### Scanning electron microscopy (SEM)

Corneas were removed at 6, 12, and 24 h after the epithelial injury from WBB6F1^+/+^ and W/W^v^ mice for scanning electron microscopy (SEM). The corneas were pre-fixed in 2.5% glutaraldehyde in 0.1 M cacodylate buffer (pH 7.4) for 2 h, then washed with cacodylate buffer, immersed in 1% tannic acid for 1 h, washed again with cacodylate buffer, and post-fixed with 2% osmium tetroxide in cacodylate buffer for 2 h. The corneas were then washed with distilled water, dehydrated in a graded ethanol series, and dried using the critical point drying method. The processed corneas were examined with SEM (Hitachi S-800; Hitachi, Tokyo, Japan).

### In vitro assay of BrdU incorporation and attachment of mouse corneal epithelial cells (MCECs)

Mouse corneal epithelial cells (MCECs) from WBB6F1^+/+^, -Sl/Sl^d^, and -W/W^v^ mice were cultured as described [35]. The MCECs were in low-bovine pituitary extract medium CnT-50 (CELLnTEC, Bern, Switzerland), and the subconfluent cells at passage 3 (P3) were incubated in a growth factor starvation medium, CnT-20 (CELLnTEC), supplemented with 5 µg/ml of insulin (Invitrogen, Inc., Tokyo, Japan) for 24 h. After the growth factor starvation, MCECs were trypsinized into single cells for the BrdU incorporation assay and the cell attachment assay.

For the BrdU incorporation assay, the MCECs were resuspended in the starvation medium supplemented or not supplemented with 10 ng/ml of rmSCF (R&D Systems, Minneapolis, MN), seeded into 24 well tissue culture plates (Asahi Techno Glass, Funabashi, Japan) at a density of 2×10^4^ cells/cm^2^, and incubated for 48 h at 37 °C under 95% humidity and 5% CO_2_. After incubation, the DNA synthetic activity of MCECs was measured with the BrdU Labeling and Detection Kit II (Roche Diagnostics, Tokyo, Japan) according to the manufacturer’s instructions. In brief, the culture medium was changed to the same fresh medium containing BrdU labeling reagent (1:1000) and incubated for 60 min at 37 °C under 95% humidity and 5% CO_2_. The MCECs were fixed in 70% ethanol buffered with 50 mM glycine buffer (pH 2, −20 °C), and then washed and incubated with a monoclonal anti-BrdU. The labeled cells were made visible by fluorescein-conjugated anti-mouse immunoglobulin (Vector Laboratories, Burlingame, CA). The nuclei were counter-stained with hoechst, and the total number of cells and the number of BrdU-positive nuclei were counted under a fluorescence microscope (IX70: Olympus, Tokyo, Japan). The BrdU incorporation is presented as the number of positively stained nuclei relative to the total number of cells.

For the cell attachment assay, the MCECs were resuspended in the starvation medium supplemented or not supplemented with 1, 10, or 30 ng/ml of rmSCF, seeded on to 96 well tissue culture plates (Corning, Inc., Corning, NY) at a density of 5×10^4^ cells/well, and incubated for 2 h at 37 °C under 95% humidity and 5% CO_2_. After incubation, the wells were washed twice with PBS(-), and the attached MCECs were trypsinized into single cells. The number of cells attached to the plates was counted using an improved Neubauer counting chamber. The number of attached cells is presented as the number of attached cells relative to the non-SCF control.

### In vitro assay of cell attachment of immortalized human corneal epithelial cells (HCECs)

Immortalized human corneal epithelial cells (HCECs) were grown to 100% confluence in supplemented hormonal epithelial medium consisting of Dulbecco's modified eagle medium (DMEM) with low glucose/F-12 (Invitrogen, Carlsbad, CA), 15% fetal bovine serum (FBS), 10 ng/ml epidermal growth factor (Invitrogen), 5 µg/ml insulin (Invitrogen), 5 mM L-glutamine (Invitrogen), 0.5% dimethyl sulfoxide (Sigma, St Louis, MO), and gentamicin (Invitrogen) [36]. All cells were grown at 37 °C in a humidified environment with 5% CO_2_. The culture medium was changed every 2 to 3 days.

To examine the involvement of SCF, the HCECs were resuspended in the starvation medium supplemented with 0, 10, 50, 250, or 1,000 ng/ml of anti-human goat SCF antibody (Santa Cruz) or control goat IgG (Santa Cruz), seeded on to 96 well tissue culture plates (Corning, Inc., Corning, NY) at a density of 5×10^4^ cells/well, and incubated for 2 h at 37 °C under 95% humidity and 5% CO_2_. After incubation, the wells were washed twice with PBS(-), and the attached HCECs were counted as described. To determine the involvement of intracellular signaling transduction by c-kit, the HCECs were resuspended in the starvation medium supplemented or not supplemented with 5, 10, 25, and 50 µg/ml of genistein (Invitrogen), a tyrosine kinase inhibitor. To examine the involvement of integrins in cell attachment, the HCECs were resuspended with or without 125, 250, 500, and 1,000 µg/ml of GRGDSP or GRGESP peptide (SIGMA-ALDRICH, Ishikari, Japan). To examine the involvement of the extracellular matrix, which has integrin binding RGD regions, the HCECs were resuspended with 1000 ng/ml of anti-human goat SCF antibodies or control goat IgG, and seeded on to tissue culture plates treated with fibronectin, laminin, or type IV collagen (Asahi Techno Glass, Funabashi, Japan). The number of attached cells is presented as the number of attached cells relative to the cells in the control.

### Statistical analyses

Statistical analyses to determine the significance of the differences between the experimental groups were performed by students’ *t* tests. The statistical significance level was set at p<0.05.

## Results

### Distribution of SCF and c-kit in ocular surface tissues

**Figure 1:**
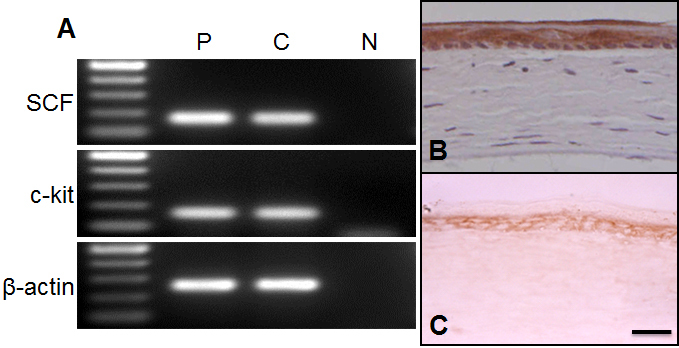
Expression of SCF and c-kit in mouse cornea. **A**: Expression of the mRNAs of *SCF* and *c-kit* in mouse cornea. Total mRNA was extracted from cornea and brain tissues of WBB6F1-^+/+^mice. The mRNAs of *SCF* and *c-kit* were detected in corneal tissue with the predicted size of approximately 170 and 160 base pairs, respectively. The brain was used as positive control (P). Negative control was carried with no DNA template (N). C:Cornea. **B**, **C**: Immunohistochemical detection of SCF and c-kit in mouse cornea. Corneal tissues from WBB6F1-^+/+^ were stained with anti-mouse SCF rabbit IgG (**B**) or anti-mouse c-kit rabbit IgG (**C**). In the cornea, a staining for SCF and c-kit was observed throughout the epithelium. Bar: 50 µm.

To determine whether SCF and c-kit were present in the cornea, we performed RT–PCR and immunohistochemistry on corneas obtained from WBB6F1^+/+^ mice. Both SCF and c-kit mRNAs were detected in the corneal tissue (Figure 1A). Immunohistochemistry showed that SCF was strongly expressed uniformly in the epithelia cells (Figure 1B), and c-kit was also expressed corneal epithelia, especially in the basal cells (Figure 1C). The c-kit receptor was expressed in both the central and peripheral cornea.

### Corneal epithelial wound closure in SCF- and C-kit mutant mice

**Figure 2:**
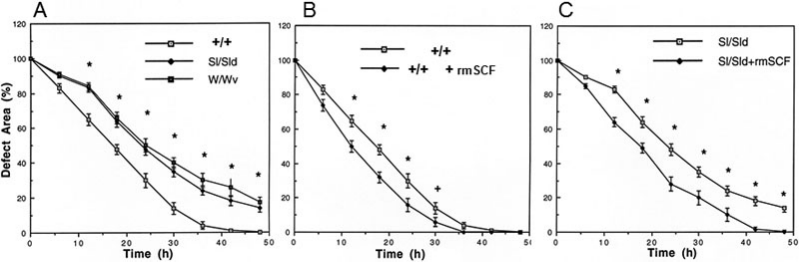
Corneal epithelial wound healing of W/W^v^- and Sl/Sl^d^ mutant mice. A 2 mm corneal epithelial defect was made, and degree of recovery was measured every 6 h. Compared to WBB6F1^+/+^ mice, the epithelial wound healing rates of both c-kit mutant mice and SCF mutant mice are significantly delayed (**A**). To determine the effect of topical rmSCF application on corneal epithelial wound healing, 5 µl of rmSCF (8 ng/ml) was applied topically to the wounded cornea every 6 h in WBB6F1^+/+^ (**B**) and SCF mutant mice (**C**). The difference in the percentage of recovery is significant between WBB6F1^+/+^ (**B**) and SCF mutant (**C**) mice. +/+=WBB6F_1_^+/+^ mice, W/W^v^=c-kit mutant mice, Sl/Sl^d^=SCF mutant mice. The error bars represent the standard errors (SE) from three experiments. *p<0.01 (paired students’ *t* test) against WBB6F1^+/+^ mice or non-administrated controls.

We examined the speed of corneal epithelial wound healing in ligand- or receptor-deficient mutant mice. The rate of wound healing in the ligand-deficient (Sl/Sl^d^) mice and the receptor-deficient (W/W^v^) mice was significantly delayed compared to that of the control WBB6F1^+/+^ mice (Figure 2A). The delay was significant even at 12 h after the epithelial injury when the Sl/Sl^d^ was 49% and the W/W^v^ mice was 47% of that of the controls (both p<0.01). In control WBB6F1^+/+^ mice, the wounds were almost completely closed at 42 h, while wound closure was 22% in the Sl/Sl^d^ mice and 28.5% in the W/W^v^ mice.

### Topical administration of recombinant SCF on wound healing of cornea

To determine whether topical application of SCF will enhance corneal wound healing, we applied recombinant SCF topically to the injured cornea of WBB6F1^+/+^ and Sl/Sl^d^ mice. The healing speed was accelerated by topical SCF in both WBB6F1^+/+^ and Sl/Sl^d^ mice within 12 h after the application (Figure 2B,C). The healing speed was especially prominent from the initial application up to 12 h after the SCF eyedrops; the residual defect area was 49% (untreated 64%) for WBB6F1^+/+^ mice and 63% (untreated 83%) for Sl/Sl^d^ at 12 h after debridement.

### BrdU incorporation during wound healing

**Figure 3:**
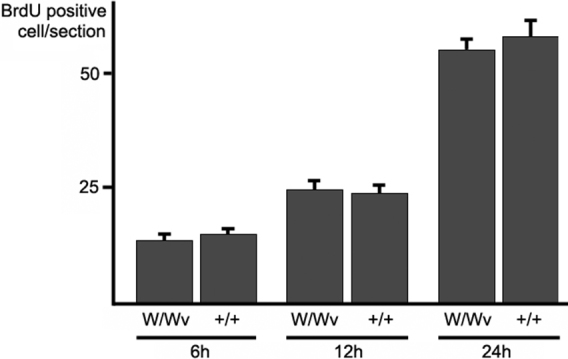
In vivo assay of BrdU incorporation during corneal epithelial wound healing. BrdU-positive corneal epithelial cells were counted in sections of WBB6F1^+/+^ and c-kit mutant corneas. Although the number of BrdU incorporated cells gradually increased after the injury, there were no significant differences between WBB6F1^+/+^ and c-kit mutant mice at each time point (unpaired *t* tests). +/+=WBB6F1^+/+^ mice, W/W^v^=c-kit mutant mice. Error bars represent the standard errors (n=6).

To determine whether the SCF/c-kit pathway enhances corneal epithelial cell proliferation during wound healing, the incorporation of BrdU by epithelial cells was determined during wound healing in WBB6F1^+/+^ and W/W^v^ mice. The number of BrdU-positive cells was significantly increased 24 h after excimer laser injury in WBB6F1^+/+^ (57.5±3.4 cells/section, n=6) and in W/W^v^ (54.6±2.3 cells/section, n=6). The difference in the number of labeled cells was not significant between the two genotypes (Figure 3).

### Scanning electron microscopy

**Figure 4:**
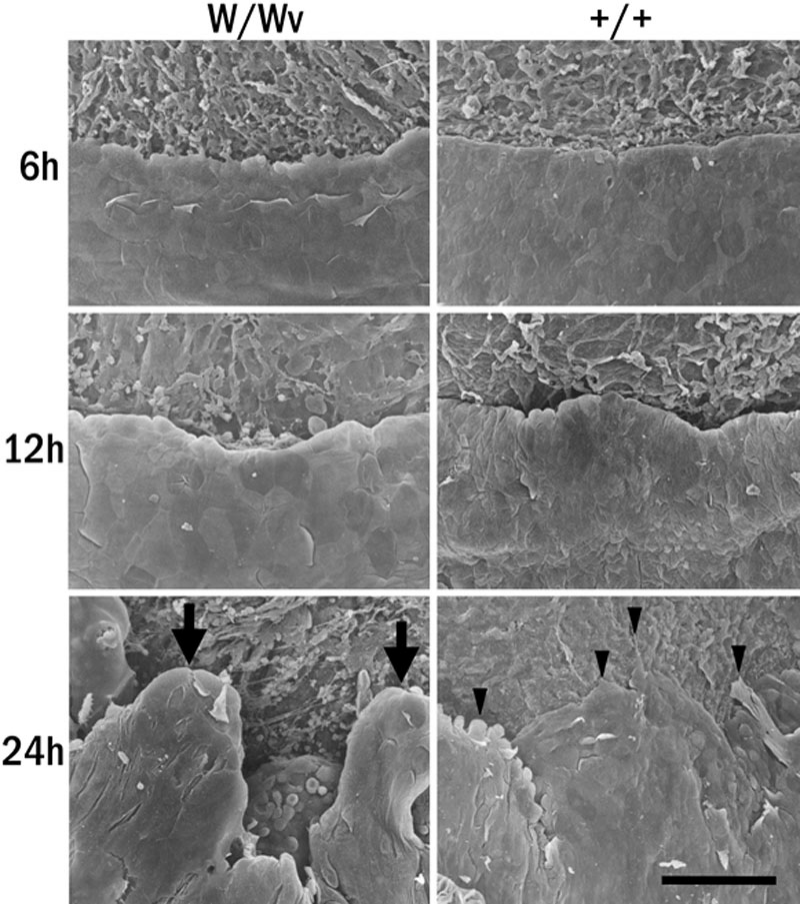
Scanning electron microscopy. The leading edges of the migrating cells of corneas from c-kit mutant and control WBB6F1^+/+^ mice, which were collected at 6, 12, and 24 h after epithelial wound healing, were examined by scanning electron microscopy. The leading edge of the c-kit mutant corneal epithelium at 24 h is elevated (arrows), whereas that of the WBB6F1^+/+^ makes flat lamellipodia (arrowheads) attachments on the cornea stroma. +/+=WBB6F1^+/+^ mice, W/W^v^=c-kit mutant mice. Bar: 100 µm.

Scanning electron microscopy was used to examine the leading edges of the corneal epithelial cells during wound closure in WBB6F1^+/+^ and W/W^v^ mice (Figure 4). The difference between the WBB6F1^+/+^ and W/W^v^ mice was clearly seen in the images at 24 h after epithelial injury. There were loosened cells in the W/W^v^ mouse cornea at 24 h after wounding, while the leading edge of WBB6F1^+/+^ mice cornea appeared to be firmly attached to the basement membrane.

### In vitro assay of BrdU incorporation

**Figure 5:**
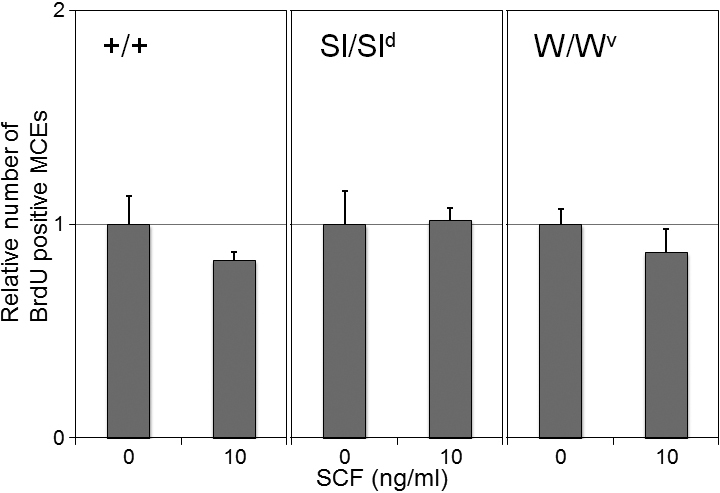
In vitro assay of BrdU incorporation. The BrdU incorporation rates were examined with rmSCF-stimulated MCECs from WBB6F1^+/+^, SCF mutant, and c-kit mutant mice. The BrdU incorporation rates are normalized to the number of non-stimulated controls (0 ng/ml rmSCF). Error bars represent the standard error (n=4). There were no significant differences between stimulated cells and non-stimulated controls (p>0.05, Man-Whitney U test). +/+=WBB6F1^+/+^ mice, W/W^v^=c-kit mutant mice, Sl/Sl^d^=SCF mutant mice.

To confirm the effect of SCF on the proliferation of the mouse corneal epithelial cells (MCECs), the incorporation of BrdU in the cultured MCECs was measured with or without addition of rmSCF to the culture medium. As in the in vivo assay, exposure to rmSCF did not accelerate the DNA synthetic activities in the WBB6F1^+/+^, SCF mutant Sl/Sl^d^, and c-kit mutant W/W^v^ mice (Figure 5). The BrdU-positive cells in the rmSCF-stimulated cultures from WBB6F1^+/+^, Sl/Sl^d^, and W/W^v^ mice were 83%, 102%, and 87% of the non-stimulated controls, respectively. There was no significant difference in these values between the rmSCF-stimulated cells and the non-stimulated controls (p>0.05, Man-Whitney U-test; Figure 5).

### In vitro assay of cell attachment of mouse corneal epithelial cells (MCECs)

**Figure 6:**
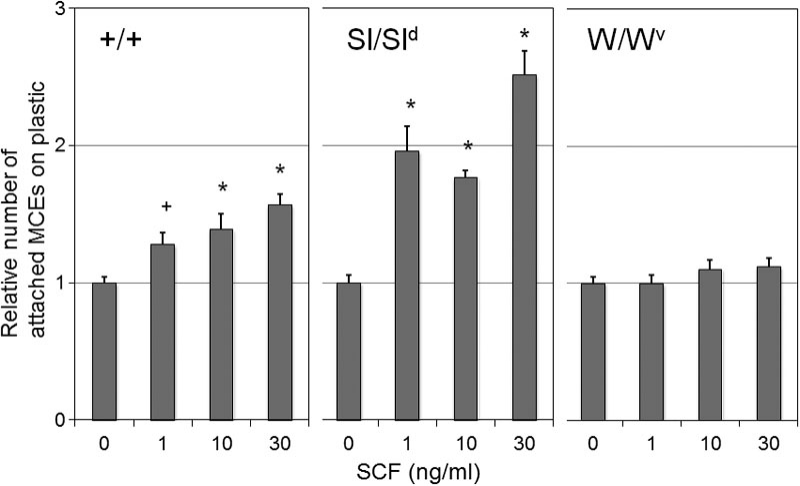
In vitro assay of attachment mouse corneal epithelial cells (MCECs). The effect of the rmSCF on the attachment of MCECs from WBB6F1^+/+^, SCF mutant, and c-kit mutant mice was examined. The MCECs were stimulated with or without 1, 10, or 30 ng/ml of rmSCF, and the results are normalized to the number of non-stimulated controls (0 ng/ml SCF). Error bars represent the standard errors (n=8). *p<0.01, ^+^p<0.05 (Man-Whitney U test) against non-stimulated controls. +/+=WBB6F1^+/+^ mice, W/W^v^=c-kit mutant strain, Sl/Sl^d^=SCF mutant strain.

Exposure to rmSCF significantly enhanced the attachment of MCECs from WBB6F1^+/+^ mice to the culture plates. With 1, 10, and 30 ng/ml of rmSCF, the percentages of attached cells were 128%, 139%, and 157%, respectively, of the non-stimulated control (Figure 6). rmSCF exposure also enhanced the attachment of cells from SCF mutant Sl/Sl^d^ mice, and the attached cells that were stimulated increased from 177% to 252% of the control (Figure 6). On the other hand, rmSCF had no influence on the attachment of c-kit mutant cells from W/W^v^ mice; the attached percentage ranged from 100% to 112% of the control, which was not significantly different from that of the non-stimulated control (Figure 6).

### In vitro assay of attachment of human corneal epithelial cells (HCECs)

**Figure 7:**
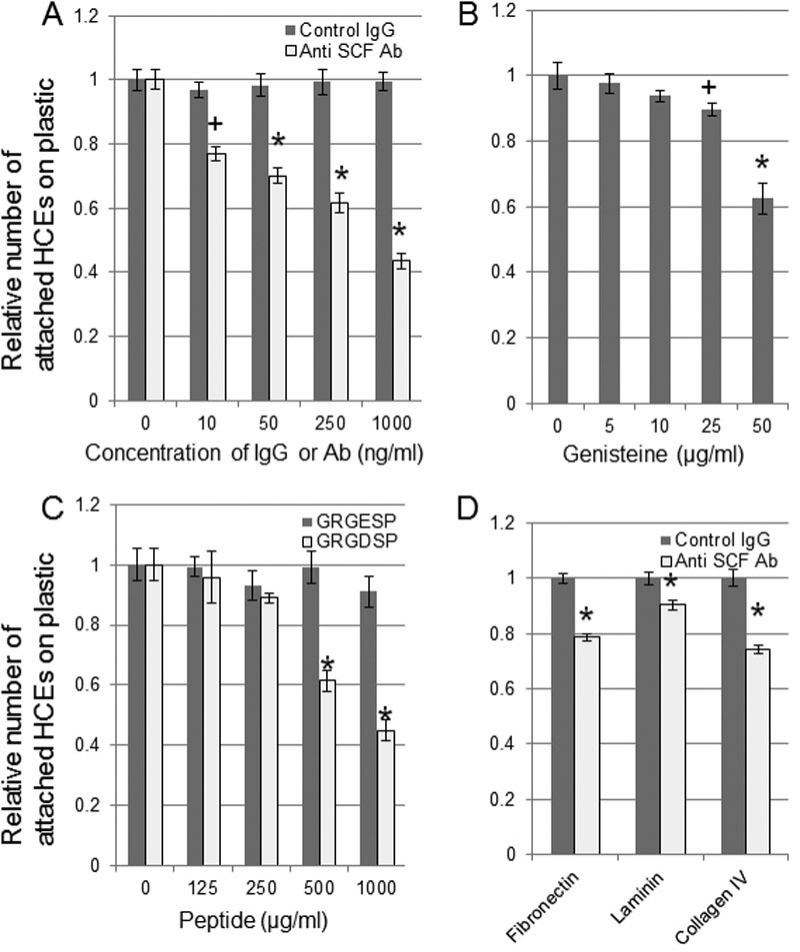
In vitro assay of attachment of human corneal epithelial cells (HCECs). The effect of anti-SCF antibodies, tyrosine kinase inhibitor genistein, RGD peptide, and extracellular matrix on the attachment of HCECs was examined. The HCECs were incubated with 0, 10, 50, 250, or 1,000 ng/ml of anti-SCF antibodies (**A**), 0, 5, 10, 25, or 50 µg/ml of genistein (**B**), or 0, 125, 250, 500, or 1,000 µg/ml of GRGDSP or GRGESP peptides (**C**). The HCECs were seeded on fibronectin, laminin, or type IV collagen-coated dishes incubated with 1,000 ng/ml of anti-SCF antibodies (**D**). The results are normalized to the number of controls. Error bars represent the standard errors (n=3). *p<0.01, ^+^p<0.05 (Man-Whitney U test) against controls.

Anti-SCF antibodies significantly reduce the percentage of attached HCECs. The percentages of attached cells that were incubated with 10, 50, 250, or 1,000 ng/ml of anti-SCF antibodies were 76.9%, 69.9%, 61.5%, and 43.6%, respectively, of the control (Figure 7A). The tyrosine kinase inhibitor genistein reduced the percentage of attached HCECs. The numbers of attached cells that were incubated with 25 and 50 µg/ml of genistein were significantly reduced to 89.5% and 62.5%, respectively, of the control (Figure 7B). The RGD peptide also reduced the percentage of HCECs attached. The attached cells that were incubated with 500 and 1000 µg/ml of GRGDSP peptide were also significantly reduced to 61.5% and 45.0%, respectively, of the control (Figure 7C). We further examined the involvement of SCF in ECMs that have RGD regions. The anti-SCF antibodies significantly reduced the attachment of HCECs to the culture plates treated with fibronectin to 78.6%, laminin to 90.3%, and type IV collagen to 74.4% of the control, respectively (Figure 7D).

## Discussion

Our results showed that both SCF and c-kit were expressed in normal mouse corneal epithelial cells, and that corneal epithelial wound healing was impaired in both Sl/Sl^d^ and W/W^v^ mice. The impaired corneal epithelial wound healing process recovered after topical application of SCF to Sl/Sl^d^ mice, a SCF mutant. These in vivo results suggested that the SCF/c-kit system is involved in corneal epithelial wound healing.

To determine by what mechanism the SCF/c-kit system enhanced corneal epithelial wound healing, we first examined whether this system stimulated the proliferation of the epithelial cells. No significant difference was found in BrdU incorporation between the wild type and W/W^v^ mice. This indicated that the absence of the SCF/c-kit system does not affect the proliferation of corneal epithelial cells.

We then examined the morphological changes in mutant mice by SEM in vivo. The SEM results showed that the corneal epithelial cells at the leading edge of the wound in W/W^v^ mouse were not stretched and did not attach to the basement membrane compared to wild type mouse. These results suggested that the absence of the SCF/c-kit system may retard the attachment of the cells to the basement membrane.

Several growth factors and membrane receptors that are involved in cell migration and cell attachment have been identified. These growth factors have been shown to regulate the healing of the injured corneal epithelium [37-42]. Nishida et al. [43,44] reported that EGF stimulates corneal epithelial cell attachment to fibronectin. Later, insulin-like growth factor-1 was shown to promote corneal epithelial cell migration and extracellular matrices production [45,46]. More recently, it was demonstrated that HB-EGF was more closely involved in promoting cell migration by cell attachments than by cell proliferation [41].

The results of our in vivo experiments suggested that the SCF/c-kit system is most likely involved in cell migration and cell attachment during corneal epithelial wound healing. The most effective method of determining the exact mechanism would be to compare the growth factors in isolated cells from wild type and SCF- and c-kit-deficient mice. Thus, we isolated the corneal epithelial cells from WBB6F1^+/+^, Sl/Sl^d^ and W/W^v^ mice by our technique [35] and examined the incorporation of BrdU and cell attachment.

As expected, enhanced cell attachment was observed in MCECs from wild type and Sl/Sl^d^ but not in W/W^v^ mice. In addition, no significant difference was detected between cells from wild type and from Sl/Sl^d^ or W/W^v^ mice in BrdU incorporation in in vitro experiments.

Thus, the results of the in vivo and in vitro experiments indicated that the SCF/c-kit system is probably involved in the attachment of corneal epithelial cells and not cell proliferation during wound healing. Although the function of the SCF/c-kit system in corneal epithelia has not been determined, it has been well shown that the SCF/c-kit system plays an important role in mast cell and progenitor cell attachment to the extracellular matrix [47-52]. In the epidermis, it has been recognized that SCF/c-kit system regulates melanocyte migration, differentiation, and proliferation [53,54]. In the cornea, it has been well documented that there is an interaction of cell adhesion molecules and ECMs controlled by other growth factors during epithelial wound healing [41,45,55-58]. It has also been demonstrated that the α6β1 integrin expression was upregulated by HB-EGF during corneal epithelial wound healing [41]. Thus, to investigate which cell adhesion molecules are regulated by SCF during corneal epithelial wound healing, we examined 88 cell adhesion-related molecules by real-time PCR using Primer Array^®^ in an in vivo wound healing model. Unfortunately, the expression of none of the molecules examined was found to be changed significantly. Recently, Trusolino et al. [59] proposed that growth factors could change the functional state of integrins from inactive to acquired ligand-binding ability with no changes of the integrin expression. They further demonstrated that hepatocyte growth factor/scatter factor (HGF/SF) induced aggregation and triggering of efficient ligand-binding capability of integrin αvβ3 without changing the expression in human thyroid cells [59]. Shimizu et al. [60] also demonstrated that the SCF/c-kit system regulates the adhesion of intestinal epithelial cells to basement membrane matrix by fibronectin receptor, VLA-5, and fibronectin interactions. They also suggested that the adhesion was caused by a change in the avidity or quality of VLA-5 rather than an increase in the density of VLA-5. The authors mentioned that the changes in the growth factor-induced avidity or affinity of integrins were regulated by ligand-receptor signaling pathways. Thus, Simizu et al. [60] demonstrated that genistein, a tyrosine kinase inhibitor, significantly inhibited the adhesion of intestinal epithelial cells to fibronectin through VLA-5. Therefore, we further examined whether the SCF/c-kit cell signaling pathway was involved in the attachment of human corneal epithelial cells, using HCECs for a cell attachment assay. As expected, anti-SCF Ab and genistein significantly inhibited the attachment of HCECs, indicating that cell adhesion was induced by the c-kit signaling pathways following SCF/c-kit interaction. The reduction induced by RGP peptide indicated the involvement of integrin-ECM interactions on the adhesion of HCECs. Additional experiments demonstrated that SCF/c-kit system might enhance the avidity or affinity of integrins to adhere to fibronectin, laminin, and type IV collagen. Although we could not determine the particular type of integrin pairs involved in the cell attachment, our results suggest that the SCF/c-kit system may be involved in the cell attachment during corneal wound healing by the induction of the avidity or affinity by one or several members of the integrin families. To determine the precise mechanisms, further investigations will be needed.

In conclusion, our results showed that SCF and c-kit were expressed in normal mouse cornea, and they provided evidence that the SCF/c-kit system plays a role in corneal wound healing by altering epithelial cell attachment.

## References

[1] Anderson DM, Lyman SD, Baird A, Wignall JM, Eisenman J, Rauch C, March CJ, Boswell HS, Gimpel SD, Cosman D, Williams DE (1990). Molecular cloning of mast cell growth factor, a hematopoietin that is active in both membrane bound and soluble forms.. Cell.

[2] Williams DE, Eisenman J, Baird A, Rauch C, Van Ness K, March CJ, Park LS, Martin U, Mochizuki DY, Boswell HS, Burgess GS, Cosman D, Lyman SD (1990). Identification of a ligand for the c-kit proto-oncogene.. Cell.

[3] Zsebo KM, Wypych J, McNiece IK, Lu HS, Smith KA, Karkare SB, Sachdev RK, Yuschenkoff VN, Birkett NC, Williams LR, Satyagal VN, Tung W, Bosselman RA, Mendiaz EA, Langley KE (1990). Identification, purification, and biological characterization of hematopoietic stem cell factor from buffalo rat liver-conditioned medium.. Cell.

[4] Martin FH, Suggs SV, Langley KE, Lu HS, Ting J, Okino KH, Morris CF, McNiece IK, Jacobsen FW, Mendiaz EA, Birkett NC, Smith KA, Johnson MJ, Parker VP, Flores JC, Patel AC, Fisher EF, Erjavec HO, Herrera CJ, Wypych J, Sachdev RK, Pope JA, Leslie I, Wen D, Lin C, Cupples RL, Zsebo KM (1990). Primary structure and functional expression of rat and human stem cell factor DNAs.. Cell.

[5] Qiu FH, Ray P, Brown K, Barker PE, Jhanwar S, Ruddle FH, Besmer P (1988). Primary structure of c-kit: relationship with the CSF-1/PDGF receptor kinase family-oncogenic activation of v-kit involves deletion of extracellular domain and C-terminus.. EMBO J.

[6] Ashman LK (1999). The biology of stem cell factor and its receptor c-kit.. Int J Biochem Cell Biol.

[7] Kent D, Copley M, Benz C, Dykstra B, Bowie M, Eaves C (2008). Regulation of hematopoietic stem cells by the steel factor/KIT signaling pathway.. Clin Cancer Res.

[8] Liu K (2006). Stem cell factor (SCF)-kit mediated phosphatidylinositol 3 (PI3) kinase signaling during mammalian oocyte growth and early follicular development.. Front Biosci.

[9] Smith MA, Court EL, Smith JG (2001). Stem cell factor: laboratory and clinical aspects.. Blood Rev.

[10] McNiece IK, Briddell RA (1995). Stem cell factor.. J Leukoc Biol.

[11] Ali S, Ravindranath N, Jia MC, Musto N, Tsujimura T, Kitamura Y, Dym M (1996). Expression of multiple c-kit receptor messenger ribonucleic acid transcripts during postnatal development of the rat testis.. Biochem Biophys Res Commun.

[12] Matsui Y, Zsebo KM, Hogan BL (1990). Embryonic expression of a haematopoietic growth factor encoded by the Sl locus and the ligand for c-kit.. Nature.

[13] Yarden Y, Kuang WJ, Yang-Feng T, Coussens L, Munemitsu S, Dull TJ, Chen E, Schlessinger J, Francke U, Ullrich A (1987). Human proto-oncogene c-kit: a new cell surface receptor tyrosine kinase for an unidentified ligand.. EMBO J.

[14] Nishikawa S, Kusakabe M, Yoshinaga K, Ogawa M, Hayashi S, Kunisada T, Era T, Sakakura T (1991). In utero manipulation of coat color formation by a monoclonal anti-c-kit antibody: two distinct waves of c-kit dependency during melanocyte development.. EMBO J.

[15] Yoshinaga K, Nishikawa S, Ogawa M, Hayashi S, Kunisada T, Fujimoto T (1991). Role of c-kit in mouse spermatogenesis: identification of spermatogonia as a specific site of c-kit expression and function.. Development.

[16] Galli SJ (1990). New insights into “the riddle of the mast cells”: microenvironmental regulation of mast cell development and phenotypic heterogeneity.. Lab Invest.

[17] Parrott JA, Kim G, Skinner MK (2000). Expression and action of kit ligand/stem cell factor in normal human and bovine ovarian surface epithelium and ovarian cancer.. Biol Reprod.

[18] Huttunen M, Naukkarinen A, Horsmanheimo M, Harvima IT (2002). Transient production of stem cell factor in dermal cells but increasing expression of Kit receptor in mast cells during normal wound healing.. Arch Dermatol Res.

[19] Peters EM, Maurer M, Botchkarev VA, Jensen K, Welker P, Scott GA, Paus R (2003). Kit is expressed by epithelial cells in vivo.. J Invest Dermatol.

[20] Botchkareva NV, Khlgatian M, Longley BJ, Botchkarev VA, Gilchrest BA (2001). SCF/c-kit signaling is required for cyclic regeneration of the hair pigmentation unit.. FASEB J.

[21] Ren X, Hu B, Colletti L (2008). Stem cell factor and its receptor, c-kit, are important for hepatocyte proliferation in wild-type and tumor necrosis factor receptor-1 knockout mice after 70% hepatectomy.. Surgery.

[22] Mouriaux F, Chahud F, Maurage CA, Malecaze F, Labalette P (2001). Implication of stem cell factor in the proliferation of choroidal melanocytes.. Exp Eye Res.

[23] Nakagami T, Murakami A, Okisaka S, Ebihara N (1997). Pterygium and mast cells–mast cell number, phenotype, and localization of stem cell factor.. Nippon Ganka Gakkai Zasshi.

[24] Kociok N, Heppekausen H, Schraermeyer U, Esser P, Thumann G, Grisanti S, Heimann K (1998). The mRNA expression of cytokines and their receptors in cultured iris pigment epithelial cells: a comparison with retinal pigment epithelial cells.. Exp Eye Res.

[25] Giebel LB, Strunk KM, Holmes SA, Spritz RA (1992). Organization and nucleotide sequence of the human KIT (mast/stem cell growth factor receptor) proto-oncogene.. Oncogene.

[26] Anderson DM, Williams DE, Tushinski R, Gimpel S, Eisenman J, Cannizzaro LA, Aronson M, Croce CM, Huebner K, Cosman D, Lyman AD (1991). Alternate splicing of mRNAs encoding human mast cell growth factor and localization of the gene to chromosome 12q22-q24.. Cell Growth Differ.

[27] Chabot B, Stephenson DA, Chapman VM, Besmer P, Bernstein A (1988). The proto-oncogene c-kit encoding a transmembrane tyrosine kinase receptor maps to the mouse W locus.. Nature.

[28] Huang E, Nocka K, Beier DR, Chu TY, Buck J, Lahm HW, Wellner D, Leder P, Besmer P (1990). The hematopoietic growth factor KL is encoded by the Sl locus and is the ligand of the c-kit receptor, the gene product of the W locus.. Cell.

[29] Zsebo KM, Williams DA, Geissler EN, Broudy VC, Martin FH, Atkins HL, Hsu RY, Birkett NC, Okino KH, Murdock DC, Jacobsen FW, Langley KE, Smith KA, Takeish T, Cattanach BM, Galli SJ, Suggs SV (1990). Stem cell factor is encoded at the Sl locus of the mouse and is the ligand for the c-kit tyrosine kinase receptor.. Cell.

[30] Besmer P, Manova K, Duttlinger R, Huang EJ, Packer A, Gyssler C, Bachvarova RF (1993). The kit-ligand (steel factor) and its receptor c-kit/W: pleiotropic roles in gametogenesis and melanogenesis.. Dev Suppl.

[31] Nocka K, Tan JC, Chiu E, Chu TY, Ray P, Traktman P, Besmer P (1990). Molecular bases of dominant negative and loss of function mutations at the murine c-kit/white spotting locus: W37, Wv, W41 and W.. EMBO J.

[32] Russell ES (1979). Hereditary anemias of the mouse: a review for geneticists.. Adv Genet.

[33] Kunisada T, Yoshida H, Yamazaki H, Miyamoto A, Hemmi H, Nishimura E, Shultz LD, Nishikawa S, Hayashi S (1998). Transgene expression of steel factor in the basal layer of epidermis promotes survival, proliferation, differentiation and migration of melanocyte precursors.. Development.

[34] Motro B, van der Kooy D, Rossant J, Reith A, Bernstein A (1991). Contiguous patterns of c-kit and steel expression: analysis of mutations at the W and Sl loci.. Development.

[35] Kobayashi T Yoshioka R Shiraishi A Ohashi Y New technique for culturing corneal epithelial cells of normal mice. Mol Vis 2009 15 1589 93 19693295 PMC2728571

[36] Araki-Sasaki K, Ohashi Y, Sasabe T, Hayashi K, Watanabe H, Tano Y, Handa H (1995). An SV40-immortalized human corneal epithelial cell line and its characterization.. Invest Ophthalmol Vis Sci.

[37] Kruse FE, Tseng SC (1993). Growth factors modulate clonal growth and differentiation of cultured rabbit limbal and corneal epithelium.. Invest Ophthalmol Vis Sci.

[38] Savage CR, Cohen S (1973). Proliferation of corneal epithelium induced by epidermal growth factor.. Exp Eye Res.

[39] Wilson SE, He YG, Lloyd SA (1992). EGF, EGF receptor, basic FGF, TGF beta-1, and IL-1 alpha mRNA in human corneal epithelial cells and stromal fibroblasts.. Invest Ophthalmol Vis Sci.

[40] Wilson SE, Walker JW, Chwang EL, He YG (1993). Hepatocyte growth factor, keratinocyte growth factor, their receptors, fibroblast growth factor receptor-2, and the cells of the cornea.. Invest Ophthalmol Vis Sci.

[41] King DC, Michels KM (1957). Muscular tension and the human blink rate.. J Exp Psychol.

[42] Yoshioka R, Shiraishi A, Kobayashi T, Morita S, Hayashi Y, Higashiyama S, Ohashi Y (2010). Corneal epithelial wound healing impaired in keratinocyte-specific HB-EGF-deficient mice in vivo and in vitro.. Invest Ophthalmol Vis Sci.

[43] Nishida T, Nakamura M, Murakami J, Mishima H, Otori T (1992). Epidermal growth factor stimulates corneal epithelial cell attachment to fibronectin through a fibronectin receptor system.. Invest Ophthalmol Vis Sci.

[44] Nishida T, Nakagawa S, Awata T, Ohashi Y, Watanabe K, Manabe R (1983). Fibronectin promotes epithelial migration of cultured rabbit cornea in situ.. J Cell Biol.

[45] Lee HK, Lee JH, Kim M, Kariya Y, Miyazaki K, Kim EK (2006). Insulin-like growth factor-1 induces migration and expression of laminin-5 in cultured human corneal epithelial cells.. Invest Ophthalmol Vis Sci.

[46] Nakamura M, Chikama T, Nishida T (1998). Up-regulation of integrin alpha 5 expression by combination of substance P and insulin-like growth factor-1 in rabbit corneal epithelial cells.. Biochem Biophys Res Commun.

[47] Dastych J, Metcalfe DD (1994). Stem cell factor induces mast cell adhesion to fibronectin.. J Immunol.

[48] Dastych J, Taub D, Hardison MC, Metcalfe DD (1998). Tyrosine kinase-deficient Wv c-kit induces mast cell adhesion and chemotaxis.. Am J Physiol.

[49] Kinashi T, Springer TA (1994). Steel factor and c-kit regulate cell-matrix adhesion.. Blood.

[50] Papayannopoulou T, Priestley GV, Nakamoto B (1998). Anti-VLA4/VCAM-1-induced mobilization requires cooperative signaling through the kit/mkit ligand pathway.. Blood.

[51] Kovach NL, Lin N, Yednock T, Harlan JM, Broudy VC (1995). Stem cell factor modulates avidity of alpha 4 beta 1 and alpha 5 beta 1 integrins expressed on hematopoietic cell lines.. Blood.

[52] Lorentz A, Schuppan D, Gebert A, Manns MP, Bischoff SC (2002). Regulatory effects of stem cell factor and interleukin-4 on adhesion of human mast cells to extracellular matrix proteins.. Blood.

[53] Jeon S, Kim NH, Kim JY, Lee AY (2009). Stem cell factor induces ERM proteins phosphorylation through PI3K activation to mediate melanocyte proliferation and migration.. Pigment Cell Melanoma Res.

[54] Takano N, Kawakami T, Kawa Y, Asano M, Watabe H, Ito M, Soma Y, Kubota Y, Mizoguchi M (2002). Fibronectin combined with stem cell factor plays an important role in melanocyte proliferation, differentiation and migration in cultured mouse neural crest cells.. Pigment Cell Res.

[55] Zhang W, Shiraishi A, Suzuki A, Zheng X, Kodama T, Ohashi Y (2004). Expression and distribution of tissue transglutaminase in normal and injured rat cornea.. Curr Eye Res.

[56] Stepp MA (2006). Corneal integrins and their functions.. Exp Eye Res.

[57] Song QH, Singh RP, Trinkaus-Randall V (2001). Injury and EGF mediate the expression of alpha6beta4 integrin subunits in corneal epithelium.. J Cell Biochem.

[58] Vigneault F, Zaniolo K, Gaudreault M, Gingras ME, Guerin SL (2007). Control of integrin genes expression in the eye.. Prog Retin Eye Res.

[59] Trusolino L, Serini G, Cecchini G, Besati C, Ambesi-Impiombato FS, Marchisio PC, De Filippi R (1998). Growth factor-dependent activation of alphavbeta3 integrin in normal epithelial cells: implications for tumor invasion.. J Cell Biol.

[60] Shimizu M, Minakuchi K, Tsuda A, Hiroi T, Tanaka N, Koga J, Kiyono H (2001). Role of stem cell factor and c-kit signaling in regulation of fetal intestinal epithelial cell adhesion to fibronectin.. Exp Cell Res.

